# Colonoscopy assisted laparoscopic sigmoidectomy: a case report

**DOI:** 10.4076/1757-1626-2-6714

**Published:** 2009-06-29

**Authors:** Satoru Takayama, Hiromitsu Takeyama

**Affiliations:** Department of Gastroenterological Surgery, Nagoya City University Graduate School of Medical Sciences1 Kawasumi, Mizuho-cho, Mizuho-ku, Nagoya 467-8601Japan

## Abstract

**Introduction:**

We report a case of colonoscopy-assisted laparoscopic sigmoidectomy used for the management of sigmoid volvulus.

**Case presentation:**

We report a 68-year-old female who underwent colonoscopy assisted laparoscopic sigmoidectomy. In this procedure, an anvil is inserted into the anus with colonoscopic assistance. An anastomosis is established without removing the colon from the abdominal cavity, and the maximum incision size is approximately 2 cm, similar to that in laparoscopic cholecystectomy. The risk of infection is lower compared with pure laparoscopic surgery, in which an incision is made for extracting the tissue specimen without opening the colon within the abdominal cavity to maintain anastomosis. The patient was discharged 1 week after surgery without complications.

**Conclusions:**

We believe that this new technique is a feasible approach for the treatment of benign lesions, particularly sigmoid volvulus, which is generally large enough to allow insertion of the anvil.

## Introduction

Laparoscopic techniques have become indispensable tools for surgeons. In colonic surgery, laparoscopic procedures are widely performed. Typically, the surgeon withdraws the colon from the abdominal cavity, resects the lesion, attaches an anvil to the oral end of the colon, returns the colon to the abdominal cavity, and then performs an anastomosis of the remaining intestine. This procedure requires an incision larger than 5 cm. Pure laparoscopic anastomosis, in which the colon is not removed from the abdominal cavity, is very difficult to perform. Therefore, laparoscopy often provides no clear advantage over open colectomy. However, in our technique, the maximum incision size is approximately 2 cm, an obvious advantage over classic laparoscopic surgery.

## Case presentation

A 68-year-old yellow raced Japanese female had a chronic history of sigmoid volvulus with persistent constipation due to paralysis of the sigmoid colon. Sigmoidectomy is considered the best therapy for such conditions. The possibility of malignancy and other abdominal diseases was eliminated by performing colonoscopy, abdominal computed tomography, and barium enema. The day before the operation we prepared the colon using 1800 ml isotonic magnesium citrate.

**Figure 1.**
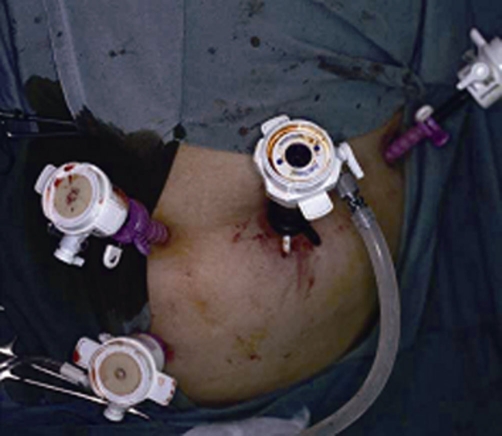
Placement of the four trocars (10, 5, 5, and 5 mm).

**Figures 2 & 3.**
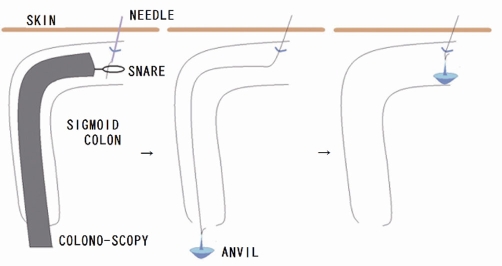

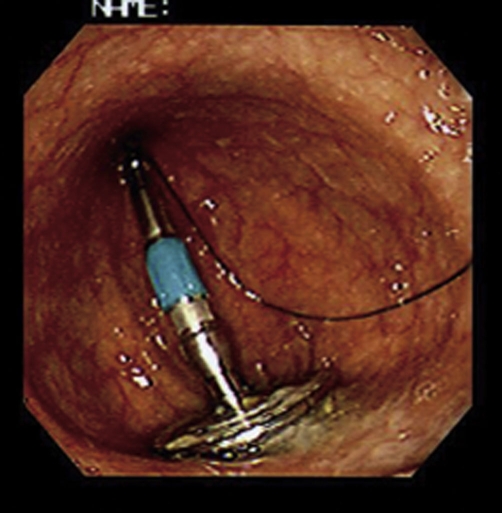
The colonoscope was inserted into the sigmoid colon through the anal aperture. An 18 G needle was inserted into the sigmoid colon percutaneously to the oral cutting point. Through this opening, a 2-0 nylon suture was inserted into the colon. It was retrieved using snare forceps and removed from the anus. The anvil was attached to it, allowing it to be pulled back to the sigmoid colon.

**Figure 4.**
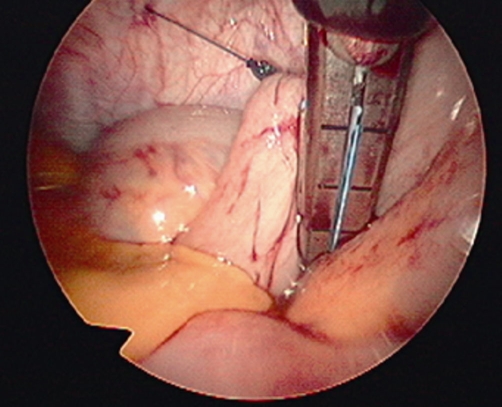
The sigmoid colon was cut with a linear stapler at a point just on the anal side of the anvil.

After inducing general anesthesia, the patient was placed in the lithotomy position. The first port (10 mm) was placed near the umbilicus, the second (5 mm) in the left upper quadrant, the third (5 mm) in the right middle flank, and the last (5 mm) in the right lower quadrant (Figure 1). After inserting the trocar, the colonoscope was inserted into the splenic flexure through the anal aperture under colonoscopic and laparoscopic monitoring. The length was sufficient to prevent the recurrence of sigmoid volvulus, which was estimated to be 40 cm in this patient. Intracolonic lavage was performed with polyvinylpyrrolidone iodine, using a colonoscope. First, an 18 G needle was inserted into the sigmoid colon to the oral cutting point. Through this opening, a 2-0 nylon suture was inserted into the colon. It was retrieved using snare forceps, and the colonoscope was removed from the anus. The anvil was attached to it, allowing it to be pulled back to the sigmoid colon (Figures 2 and 3). The linear stapler was used to cut the anal end of the anvil and the end of the sigmoid colon (Figure 4). The mesosigmoid colon was cut using laparoscopic coagulating shears. Before anastomosis, the anvil was cleaned with iodine. The rest of the colon was anastomosed from the oral side of the anvil to the anal side of the shaft. Finally, the resected sigmoid colon was removed through the umbilical incision. An incision of less than 2 cm was adequate.

**Figure 5.**
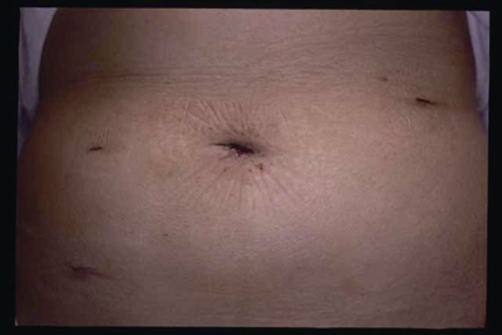
Operative scars after operation.

Two days after surgery, the patient started eating and her body temperature normalized. The following day, the patient walked comfortably with only slight abdominal pain. Her bowel function improved compared with its preoperative status. One week after surgery, the operative scar healed completely (Figure 5) and the patient was discharged with no complications.

## Discussion

Using the technique described here, the size of the operative scar is almost identical to that after laparoscopic cholecystectomy. Benign lesions, sigmoid volvulus, diverticulitis, and inflammatory bowel diseases have been treated with laparoscopic approaches, [1-4] but most of these cases were treated by a method identical to that used in malignant cases, in which the colon is anastomosed outside the abdominal cavity. Large incisions are required for this procedure. This new technique seems to be applicable to several diseases, apart from malignancies, where it is impossible to remove the colon without incising the tumor. In addition, anastomoses that require the colon to be opened in the intra-abdominal cavity, such as intra-abdominal functional end-to-end anastomosis, are also performed as colonic anastomoses [5]. However, this necessitates opening the colon in the abdominal cavity, and also requires an additional staple line compared with our method. Therefore, our method is superior to functional end-to-end anastomosis and other anastomoses for the view point of risks of infection and leakage.

## Conclusions

Several features of malignant growths, such as their size and location, may restrict the application of the method described here. However, this method is feasible in disease conditions that have a sufficiently large lumen for anvil insertion. And more this technique may be useful for minimum invasive surgery such as “NOTES” in near future.

## References

[bib-001] Bouillot JL, Aouad K, Badawy A, Alamowitch B, Alexandre JH (1998). Elective laparoscopic-assisted colectomy for diverticular disease. A prospective study in 50 patients. Surg Endosc.

[bib-002] Mooney MJ, Elliott PL, Galapon DB, James LK, Lilac LJ, O'Reilly MJ (1998). Hand-assisted laparoscopic sigmoidectomy for diverticulitis. Dis Colon Rectum.

[bib-003] Canin-Endres J, Salky B, Gattorno F, Edye M (1999). Laparoscopically assisted intestinal resection in 88 patients with Crohn's disease. Surg Endosc.

[bib-004] Lee SW, Yoo J, Dujovny N, Sonoda T, Milsom JW (2006). Laparoscopic vs. hand-assisted laparoscopic sigmoidectomy for diverticulitis. Dis Colon Rectum.

[bib-005] Sundin JA, Wasson D, McMillen MM, Ballantyne GH (1992). Laparoscopic-assisted sigmoid colectomy for sigmoid volvulus. Surg Laparosc Endosc.

